# Selective Area Epitaxy of Highly Strained InGaAs Quantum Wells (980–990 nm) in Ultrawide Windows Using Metalorganic Chemical Vapor Deposition

**DOI:** 10.3390/nano13172386

**Published:** 2023-08-22

**Authors:** Viktor Shamakhov, Sergey Slipchenko, Dmitriy Nikolaev, Alexander Smirnov, Ilya Eliseyev, Artyom Grishin, Matvei Kondratov, Ilya Shashkin, Nikita Pikhtin

**Affiliations:** Ioffe Institute, 26 Politekhnicheskaya, St. Petersburg 194021, Russia; shamakhov@mail.ioffe.ru (V.S.); dim@mail.ioffe.ru (D.N.); alex.smirnov@mail.ioffe.ru (A.S.); ilya.eliseyev@mail.ioffe.ru (I.E.); ar.evg.grishin@yandex.ru (A.G.); mikondratov99@gmail.com (M.K.); shashkin@mail.ioffe.ru (I.S.); nike@mail.ioffe.ru (N.P.)

**Keywords:** selective area epitaxy, MOCVD, quantum well, InGaAs, photoluminescence, profilometry, vapor-phase diffusion model

## Abstract

We employed the selective-area-epitaxy technique using metalorganic chemical vapor deposition to fabricate and study samples of semiconductor heterostructures that incorporate highly strained InGaAs quantum wells (980–990 nm emission wavelength). Selective area epitaxy of InGaAs quantum wells was performed on templates that had a patterned periodic structure consisting of a window (where epitaxial growth occurred) and a passive mask (where epitaxial growth was suppressed), each with a width of 100 µm for every element. Additionally, a selectively grown potential barrier layer was included, which was characterized by an almost parabolic curvature profile of the surface. We conducted a study on the influence of the curvature profile of the growth surface on the optical properties of InGaAs quantum wells and the spatial distribution of composition in an ultrawide window. Our results showed that, under fixed selective-area-epitaxy conditions, the composition of the In*_x_*Ga_1−*x*_As and the wavelength of the quantum-well emission changed across the width of the window. Our study demonstrates that increasing the curvature profile of the growth surface of highly strained quantum wells leads to a transition in the photoluminescence wavelength distribution profile across the window, from quasi-parabolic to inverted parabolic.

## 1. Introduction

Light-emitting devices (LED) based on semiconductor heterostructures are currently widely used in practice. The main types of such devices include LEDs, semiconductor lasers, and optical amplifiers. The key element of such devices is the active region. In most cases, active-region designs are based on structures with pronounced quantum size properties. The main types of such structures are quantum wells, filaments, and dots [1]. The use of quantum-well structures significantly reduces threshold currents, which is important for semiconductor lasers, and expands the range of compositions that can be used in strained structures, which is important for all types of light-emitting devices [1,2]. However, classical approaches based on standard epitaxy (SE), when the layer grows over the entire planar surface, significantly limit the design optimization and development of new structures. In particular, SE provides a uniform active-region (AR) composition over the entire structure surface. In this case, wavelength control is feasible, for example, through the use of various types of photonic crystals [3,4,5,6,7,8], including conventional Bragg gratings; however, the source efficiency and the tuning range will still be limited by the AR composition and thickness, which do not change in the case of SE [9]. This is primarily due to the photonic-crystal-operating-wavelength shift from the maximum of the material gain spectrum. At the same time, there are a number of topical tasks that require flexible control of AR spectral characteristics within a single heterostructure, implemented by local changes in the composition and thickness of the quantum-well ARs. Such tasks include the creation of photonic integrated circuits, when it is required to combine a number of active elements of various spectral properties (lasers, detectors, amplifiers, waveguides, and modulators) within a single monolithic structure [10,11,12], as well as the creation of multispectral laser sources, effectively operating in a wide spectral range, for example, for the wavelength division multiplexing [13,14,15], the formation of an absorption section for mode-locked lasers [16].

The available technique that allows flexible control of the quantum-well AR properties is a selective area epitaxy (SAE). Within the framework of this technique, growth occurs in windows on a pre-prepared surface with a dielectric mask, and growth is suppressed on the mask surface. The sizes of windows and masks allow flexible control of the composition and growth rate. As a result, structures with arrays of ARs were demonstrated, showcasing a tuning range of up to 86 nm [17] and 150 nm [18] for the maximum of the photoluminescence spectrum, achieved through varying the AR composition. Despite the achieved results, the issue of describing the basic features of SAE in the case of highly strained quantum wells (QWs) remains. The fundamental principles of SAE, in the case of bulk AlGaAs layers, were studied in [19,20]. Experimental studies of the growth mechanisms of InGaAs/GaAs quantum wells are considered in [17]. However, the SAE of the structure includes a number of aspects that have not been considered so far and are important for optimizing both the design and process parameters. To date, most studies describe the SAE of QWs in windows with a width not exceeding several tens of microns [21,22]. The results of studies of QWs obtained by the SAE in ≥ 100 µm wide windows are presented in [17,23,24,25]; however, a number of important issues related to the effect of curvature (bending) of layers obtained by SAE on the properties of QWs, as well as the possibility of describing the QW characteristics using existing simulation models, remain unexplored.

Here, we consider the SAE mechanisms of highly strained QWs in ultrawide windows. The significant effect of the lower SAE-grown waveguide layer on the radiative characteristics of InGaAs/GaAs QWs is demonstrated, the experimental results on the PL characteristics of InGaAs/GaAs QWs are analyzed within the framework of the vapor-phase diffusion model, and potential limitations in describing the growth behavior of highly strained InGaAs/GaAs QWs are identified.

## 2. SAE QW Experimental Samples and Research Technique

Experimental samples were grown by metalorganic chemical vapor deposition (MOCVD). For growth, we used an EMCORE GS3100 (EMCORE Corp., Somerset, NJ, USA) setup with a vertical reactor and resistive heating of the substrate holder. The growth temperature was 750 °C; the rotation speed of the substrate holder was 1000 rpm. Trimethylgallium (TMGa), trimethylindium (TMIn), and trimethylaluminum (TMAl) (Elma-Chem, Zelenograd, Russia) were used as group 3 reagents, and arsine (AsH3) (Salyut, Nizhny Novgorod, Russia) was used as a group 5 reagent. The carrier gas was hydrogen. Epitaxial growth was carried out on an n-GaAs (100) substrate (Wafer Technology Ltd., Milton Keynes, UK).

As part of the research, 2 sets of samples were grown. Samples of the first set were grown by SE and included a 0.5 μm thick GaAs buffer, 0.4 μm thick Al_0.3_Ga_0.7_As lower cladding, 0.8 μm thick GaAs waveguide, InGaAs QW located in the waveguide center, and 0.25 μm thick Al_0.3_Ga_0.7_As upper cladding. The first set included 2 samples that differed in the QW growth time: 7 s for the SENQW (standard epitaxy narrow QW) sample and 14 s for the SEWQW (standard epitaxy wide QW) sample. These samples were grown for the purpose of evaluating the technique, which will be further utilized for determining the composition and thickness of QWs.

The second set of samples consisted of heterostructures obtained by SAE. Samples were made in 2 stages. In the first stage, a preform was grown by SE: 0.5 μm thick GaAs buffer, 0.4 μm thick Al_0.3_Ga_0.7_As lower cladding, and 0.4 μm thick GaAs waveguide lower part. After that, 100 nm thick SiO_2_ dielectric coating was deposited on the obtained preform by reactive ion-plasma sputtering. Next, a mask pattern was formed with alternating 100 µm wide stripes (SiO_2_ mask/window) using lithography and a buffer etchant (BOE 5:1). The stripes were formed in the [011] direction. Then the second stage of sample growth using SAE began. Within the second stage, the lower part of the GaAs waveguide layer was grown onto the preforms obtained. The waveguide-layer lower-part thickness varied in the range of 0.12–1.9 µm; the conditions were the same and corresponded to the growth rate at SE equal to 19 nm/min; different thicknesses were obtained by changing the growth time. Next, the InGaAs QWs were grown by SAE. The process parameters were consistent with those of the SENQW sample growth and were the same for all SAE samples. Next, 0.3 μm thick GaAs waveguide upper part and 0.3 μm thick Al_0.11_Ga_0.89_As upper cladding were grown at the same time for all SAE samples’ growth rate, which corresponds to the growth rate of 21.3 nm/min at SE of Al_0.11_Ga_0.89_As.

The thicknesses of SAE-grown layers shown in Table 1 are measured at the window center. The samples of this set differed in the thickness of the GaAs lower waveguide part grown by SAE (SAEWG): the SAEWG1 sample was 0.12 µm, the SAEWG2 sample was 0.6 µm, the SAEWG3 sample was 1.2 µm, and the SAEWG4 sample was 1.9 µm (the description of the samples is given in Table 1). This remark is due to the fact that the lower-waveguide-layer thickness profile changes across the window width in the SAE samples. As the layer thickness increases, the difference between the layer thickness at the window center and at the window edge increases in the SAE samples. The mechanisms determining the thickness profile across the window width described in [19] for bulk GaAs layers demonstrate that a change in thickness significantly modifies the growth mode (a transition from step flow to step bunching is observed) and the structure of monoatomic steps. In addition, the layer-thickness drop between the edge and center of the window changes; in our case, it increases from 13.5 to 272 nm with the lower SAE waveguide thickness increasing from 0.12 to 1.9 µm.

**Figure 1:**
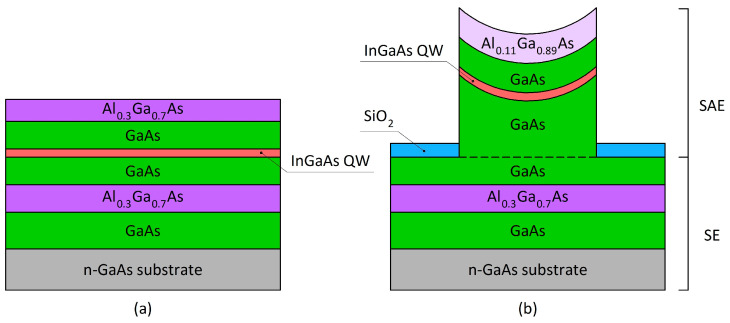
Schematic illustration of the samples under study: (**a**) the sample obtained by SE and (**b**) the sample obtained by SAE.

Figure 1 shows a schematic illustration of the samples under study: (a) the sample obtained by SE and (b) the sample obtained by SAE.

We used the following techniques in the study:

- Spatially resolved microphotoluminescence (µPL) was used to study the QW luminescence characteristics of samples from both sets. The µPL measurements were performed at room temperature using a T64000 (Horiba Jobin Yvon, Longjumeau, France) spectrometer equipped with a confocal microscope. These spectra were measured using the continuous-wave (cw) excitation at 532 nm (2.33 eV) of a Nd:YAG laser (Torus, Laser Quantum, Stockport, UK) with a power on the samples as low as ~40 µW. The spectra were recorded using a 600 lines/mm grating and liquid-nitrogen-cooled charge-coupled device (CCD) camera with the Mitutoyo 100 × NIR (NA = 0.90) long working-distance objective lens to focus the incident beam into a spot of ~2 μm diameter. The measurements were carried out with point-to-point scanning with a step of 1 μm.
- for SAEWG samples, measurements of the thickness profile across the window were carried out using an AmBios XP-1 profilometer (Ambios Technology Inc., Santa Cruz, CA, USA). To do this, the SiO_2_ mask was preliminarily removed from the samples.

## 3. Experimental and Theoretical Studies of the Characteristics of SAE QWs

**Figure 2:**
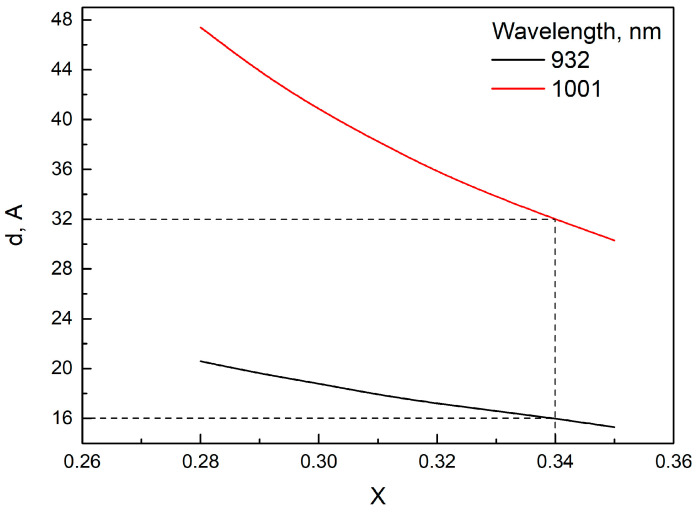
Thickness (*d*) of the In*_x_*Ga_1−*x*_As QW as a function of the composition (*x*), providing emission wavelengths of 932 and 1001 nm.

For the first set of samples, PL studies were carried out at a temperature of 300 K. The SENQW and SEWQW samples had the maximum wavelengths of the PL spectrum at 932 and 1001 nm, respectively. These samples were grown with the purpose of evaluating the characteristics of SE QWs (thickness and composition) and aligning the measurement results with calculations for analyzing the compositions and thicknesses of SAE QWs. Since a given wavelength can be obtained with QWs of different compositions and thicknesses, calculations of possible combinations of compositions and thicknesses that provide wavelengths of 932 nm and 1001 nm were carried out. These calculations were based on solving the Schrödinger equation for a rectangular QW of finite depth. Mechanical strains in the QW and band discontinuities between the QW and the waveguide were taken into account in accordance with [26]. The results are plotted in Figure 2. Next, the composition of the In*_x_*Ga_1−*x*_As QW was determined, in which the following condition is satisfied: the transition in wavelength from 932 nm to 1001 nm is achieved by doubling the QW thickness. Figure 2 shows that this condition is satisfied only by the composition of the In*_x_*Ga_1−*x*_As QW, equal to *x* = 0.34, and the QW thicknesses of the SENQW and SEWQW samples, 16 Å and 32 Å, respectively.

**Figure 3:**
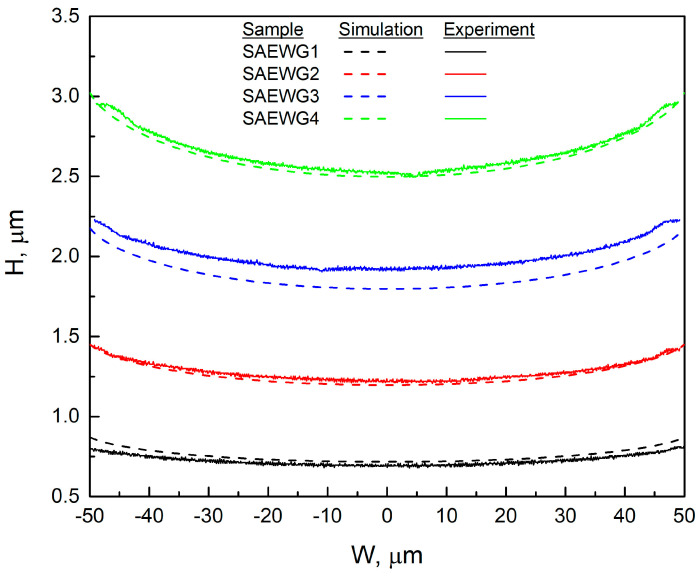
The thickness of the structure grown by SAE across the window width. Solid lines are experimental data; dotted lines represent calculated data obtained using Equation (2).

Figure 3 shows experimental thickness profiles across the window width (solid lines) for samples SAEWG1—SAEWG4 obtained by SAE. The dotted lines show the calculated total thickness profile across the width of the window. We used the growth-rate-enhancement (*GRE*) distributions for GaAs and Al_0.11_Ga_0.89_As [20] to calculate the total thickness. The SAE single-layer thickness (*H*) is determined as follows [20]:

(1) $$H=GRE\cdot V_{planar}\cdot t,$$

where *V_planar_* is the growth rate of a given material at SE and *t* is the growth time of the SAE layer.

The SAE-grown multi-layer total thickness (*H_s_*) for the SAEWG samples is determined as follows:

(2) $$H_{s}={GRE}_{GaAs}\cdot V_{GaAs}\cdot \left(t_{lw}+t_{uw}\right)+{GRE}_{AlGaAs}\cdot V_{AlGaAs}\cdot t_{uc},$$

where *GRE_GaAs_* and *GRE_AlGaAs_* are the *GREs* of the GaAs and Al_0.11_Ga_0.89_As layers, respectively [20]; *V_GaAs_* and *V_AlGaAs_* are the GaAs and Al_0.11_Ga_0.89_As layer growth rates at SE, respectively; *t_lw_*, *t_uw_*, and *t_uc_* are the growth times of the SAE layers of the lower and upper waveguides and the upper cladding, respectively (Table 1). The QW thickness was not taken into account because it is negligible compared with the thickness of the other layers.

Figure 3 demonstrates that the specified thickness values align fairly accurately with the corresponding experimental values. However, the simulation and experiment is partially inconsistent for SAEWG3 compared with the other samples. One possible reason for this behavior is a change in the growth mode, such as a transition from step flow to bunching, which can affect the growth rate. The presence of a strained QW may also enhance this effect. However, to pinpoint the factors that contribute to this behavior, further research is needed, especially surface morphology studies.

**Figure 4:**
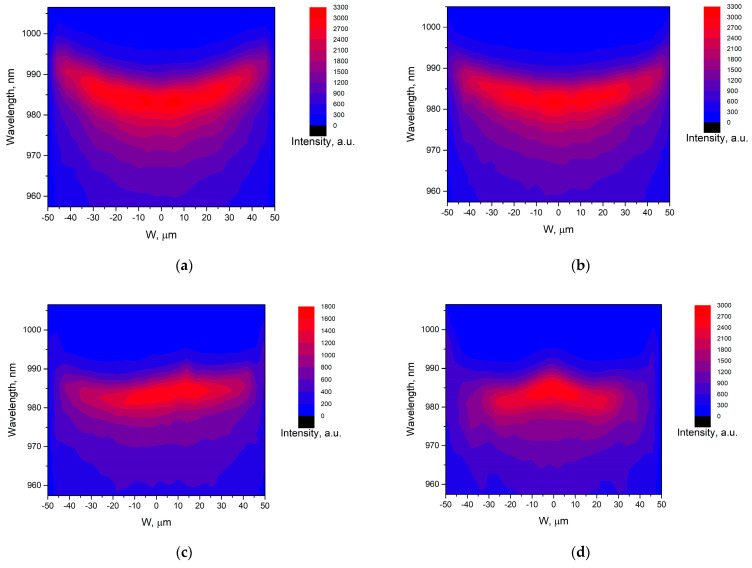
InGaAs QW μPL spectra maps across the window width at 300 K for samples: (**a**) SAEWG1, (**b**) SAEWG2, (**c**) SAEWG3, and (**d**) SAEWG4.

**Figure 5:**
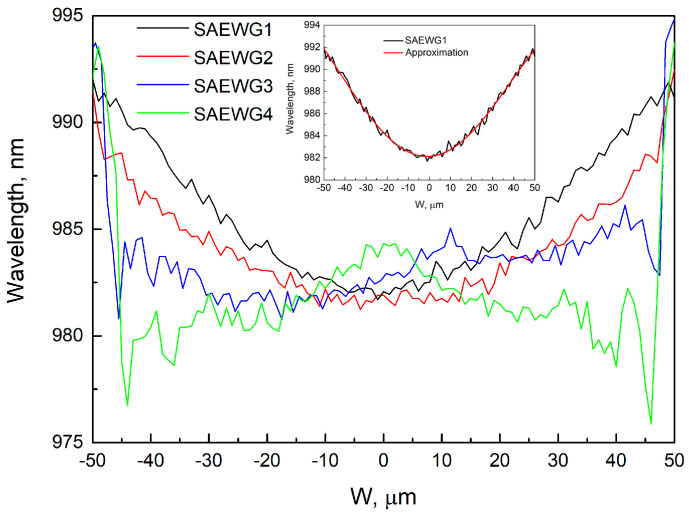
Experimental variation of the µPL peak wavelength across the window for SAEWG1—SAEWG4 samples, with a 4th-degree polynomial approximation of the experimental data for the SAEWG1 sample shown in the inset.

Figure 4a–d show the µPL spectra maps at 300 K for the SAEWG1–SAEWG4 samples. The maximum intensity is observed at the window center for all samples, whereas it decreases significantly towards the window edges. It can also be seen from Figure 4a,b that the SAEWG1 and SAEWG2 samples exhibit a gradual redshift in wavelength as one moves from the window center towards the edges. Figure 4c,d reveal a unique wavelength distribution for samples SAEWG3 and SAEWG4, with minimal change in the central region and a rapid increase in wavelength towards the window edges. To enhance the accuracy of subsequent analyses, we demonstrate the distribution of the µPL spectrum maximum across the window for all SAEWG1—SAEWG4 samples (depicted in Figure 5). According to the dependencies, a continuous increase in wavelength is observed from the center (982 nm) to the edge (992 nm) of the window for the SAEWG1 and SAEWG2 samples. In the case of the SAEWG3 sample, the wavelength undergoes a small change (a 4 nm shift) at the center of the window (−45 µm to 47 µm), followed by a sharp increase to 994 nm towards the edges. For the SAEWG4 sample, the wavelength gradually decreases from 984 nm (the window center) to 976 nm (the edges of the interval from −44 µm to 46 µm) as the distance from the center increases towards these boundaries. As the distance from the window center increases towards the window edges, the wavelength sharply increases, reaching up to 994 nm. Note that the SAEWG samples differ only in the thickness of the lower waveguide layer grown by SAE. These results suggest that the observed dependencies are affected by the changes in the curvature of the lower-waveguide-layer profile. Using Equation (1) to estimate the change in SAE layer thickness between the center and edge of the window, we find that this change increases from the SAEWG1 to SAEWG4 sample from 25 to 395 nm. Our findings indicate that the wavelength variation across the window has a similar form to that observed in [17,23], up to a thickness of 0.6 µm of the lower waveguide layer grown via SAE. For larger thicknesses, the wavelength variation across the window exhibits a different behavior.

An analysis of the variation in composition and thickness of the QW layer grown through SAE was performed next. To do this, the basic parameters of the SAE QW (composition and thickness) were calculated based on the experimental wavelength values obtained in the window (Figure 5). The vapor-phase diffusion model [20,27] was used for the simulation. The main characteristic of the deposited layer during SAE is the change in *GRE* across the window width. By using the *GRE* values of the binary compounds, it is possible to calculate the *GRE* of the resulting ternary solid solution:

(3) $${GRE}_{InGaAs}=x_{0}\cdot {GRE}_{InAs}+\left(1-x_{0}\right)\cdot {GRE}_{GaAs},$$

where *GRE_InGaAs_*, *GRE_InAs_*, and *GRE_GaAs_* are the *GREs* of InGaAs, InAs, and GaAs, respectively, and *x*_0_ are In mole fraction in the InGaAs solid solution under the same growth conditions at SE.

By applying the vapor-phase diffusion model and taking into account the composition value *x*_0_ and layer thickness *d*_0_ during SE, the changes in composition *x* and thickness *d* of the layer across the window width during SAE can be estimated:

(4) $$d={GRE}_{InGaAs}\cdot d_{0},$$

(5) $$x=\frac{x_{0}\cdot {GRE}_{InAs}}{{GRE}_{InGaAs}},$$

**Figure 6:**
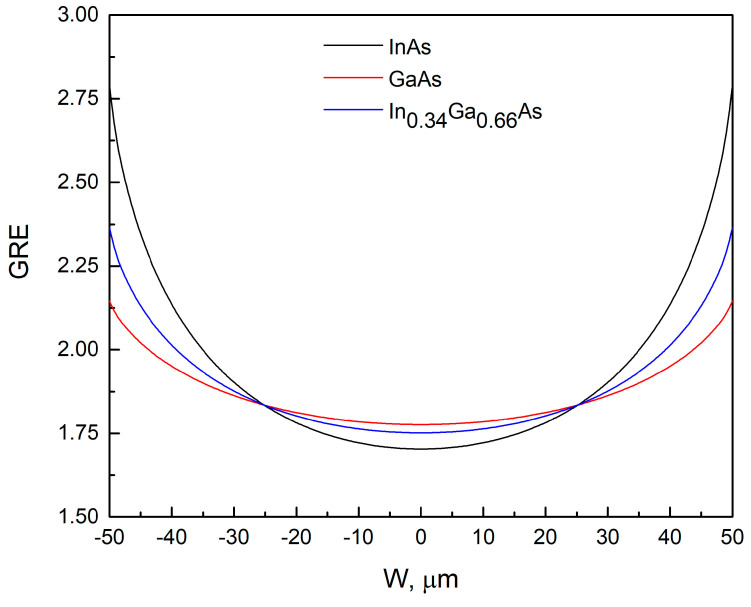
Distribution of *GRE* across the window width for *D/k* values of 85 μm for Ga and 25 μm for In.

**Figure 7:**
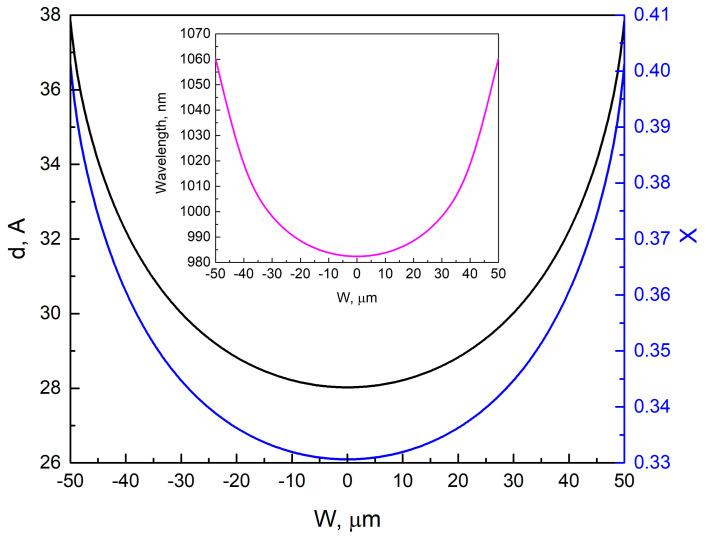
Calculated variation in thickness (*d*) and composition (*x*) of the In*_x_*Ga_1−*x*_As QW grown by SAE for D/k values of 85 μm for Ga and 25 μm for In. Inset: Emission wavelength across the window width for the QW with parameters shown in Figure 7.

In the case of a QW, the obtained values of the composition *x* and thickness *d* make it possible to calculate the wavelength for each point of the window by solving the Schrödinger equation for a rectangular QW of finite depth. When an effective diffusion length (*D/k*) of 85 µm is used for Ga within the vapor-phase diffusion model, there is good agreement between the experimental and calculated results for GaAs, as shown in [20]. In the present study, for the purpose of the QW simulation, the *D/k* value of 85 μm was chosen for Ga. The *D/k* value for In was adjusted to obtain a wavelength of 982 nm at the window center. According to [25,28], *D/k* for Ga is larger than for In. Estimates have shown that the required wavelength at the window center is obtained at *D/k* for In equal to 25 µm. Figure 6 shows the calculated values of the *GRE* across the window for GaAs, InAs, and In_0.34_Ga_0.66_As, in which composition corresponds to SE. Figure 7 shows the thickness *d* and composition *x* profiles across the window for the In*_x_*Ga_1−*x*_As QW grown via SAE under conditions corresponding to SE of a QW with thickness *d*_0_ = 16 Å and composition *x*_0_ = 0.34. The dependence of the change in QW emission wavelength across the window width can be derived from the calculated composition *x* and thickness *d* of the QW layer grown via SAE, as shown in the inset of Figure 7. It can be seen that the wavelength changes from 982 nm at the window center to 1060 nm at the window edge. Thus, with *D/k* values determined for Ga (85 μm) and In (25 µm), the change in the wavelength corresponding to the maximum of the photoluminescence spectrum is as high as 78 nm across the window width. The calculated value is significantly higher than the value obtained from experimental data, with a maximum difference of only about 10 nm in the photoluminescence wavelength between the central and outer regions of the window (refer to Figure 5). According to [17], InGaAs QWs display a pronounced wavelength redshift from the window center to the edge, which is attributed to an increase in the QW thickness and the amount of In in the In*_x_*Ga_1−*x*_As QW grown via SAE as one moves from the window center to the edge.

## 4. Discussion of the Results

**Figure 8:**
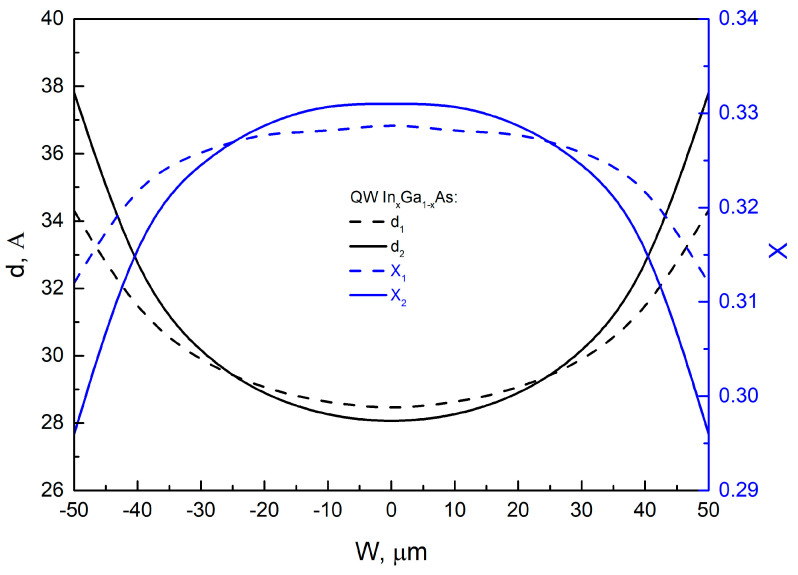
Thickness (*d*) and composition (*x*) variation in an In*_x_*Ga_1−*x*_As QW fabricated using SAE: *d*_1_, *x*_1_—approach 1; *d*_2_, *x*_2_—approach 2.

To explain the significant difference between the experimental (Figure 5) and calculated (Figure 7 inset) results for the PL wavelength change of the SAE QW across the window, further analysis was conducted. The wavelength distribution of the SAEWG1 sample (Figure 5) was selected for analysis, as it had the lowest curvature in the lower waveguide layer, which should minimize the influence of the profile and morphology on the grown SAE QW. The experimental dependence was described by a fourth-degree polynomial for ease of analysis (Figure 5 inset). It is known that the maximum PL wavelength of the QW is determined by its composition, thickness, and energy depth relative to the waveguide layer, which is also influenced by the waveguide layer’s composition. The waveguide-layer composition was kept constant in all experiments. Therefore, the composition and thickness of the QW were the primary factors that influenced the wavelength. The behavior of the maximum PL wavelength across the window for the SAE QW was analyzed using two approaches. The first approach used the *GRE* dependence for GaAs (red curve in Figure 6) to calculate the SAE QW thickness using Equation (4). This was justified by the fact that the QWs investigated in this study contained more Ga than In. However, it should be noted that the In content in the QW grown by SAE was 0.34, which is significantly higher than the 0.12 content in the QW studied in [17]. To calculate the composition variation of the SAE QW across the window, we followed a two-step process. First, at each point of the window, we obtained the thickness and emission wavelength of the QW. Then, we solved the Schrödinger equation for a rectangular QW of finite depth to determine the composition needed to produce the specified wavelength at the given thickness. As for the second approach, we relied on the GRE dependence observed for the In_0.34_Ga_0.66_As layer (Figure 6, blue curve). Using this data and a process similar to the first approach, we determined the composition of the QW across the window. Figure 8 illustrates the results obtained by both approaches, with the QW thickness (*d*) and composition (*x*) of In*_x_*Ga_1−*x*_As plotted against the window position (approach 1—the dashed curves; approach 2—the solid curves). The possible dependencies of the composition of In*_x_*Ga_1−*x*_As QWs on the window width, obtained by SAE, were calculated based on both the dependencies of QW thickness on window width, which were determined using the approaches described above (Figure 8, curves *d*_1_ and *d*_2_), and the experimental distribution of the QW emission wavelength across the window width for the SAEWG1 sample (Figure 5, inset). From Figure 8 (curves *x*_1_ and *x*_2_), it can be seen that in order to achieve the experimentally observed change in the wavelength across the window width, the QW In*_x_*Ga_1−*x*_As composition should decrease when moving from the window center to the edge as the thickness of the QW increases. It should be noted that such behavior is observed for both approaches to estimate the change in QW thickness across the window width. Within the framework of the vapor-phase diffusion model, such a behavior of the composition can occur when *D/k*(In) > *D/k*(Ga), despite the opposite *D/k* values reported for In and Ga in the literature [25,28]. The observed behavior can be attributed to the fact that under certain growth conditions, such as the growth temperature, a maximum In concentration can be achieved in the strained InGaAs QW. Once this maximum concentration is reached, any further increase in the In content in the vapor phase leads to a decrease in its concentration within the QW, as reported in [29]. According to [29], this reduction is caused by the formation of unstrained InAs islands, into which indium can incorporate without a strain-induced barrier. Some of the excess indium is desorbed from the surface of these InAs clusters during growth, and some small part is accumulated into defects, which can be identified as dislocation loops located around small regions with higher In content [29]. During SAE, the In content in the vapor phase at the window edge is higher than at the window center. This distribution is confirmed by the calculated *GRE* for In (Figure 6, black curve). However, it should be understood that the distribution across the window width of the actual thickness *d* and composition *x* of the In*_x_*Ga_1−*x*_As QW obtained by SAE lies somewhere between the values obtained within the two approximations used. In order to verify this assumption regarding the behavior of QW composition, future studies will focus on QWs with lower In content. This approach should help eliminate the influence of the maximum In incorporation factor in highly strained QWs. It can also be assumed that at high In concentrations, the curvature of the lower part of the waveguide layer obtained by SAE may affect its incorporation.

## 5. Conclusions

It has been shown that a highly strained In_0.34_Ga_0.66_As QW (with a composition corresponding to standard epitaxy) grown by selective area epitaxy on an n-GaAs (100) substrate with an alternating stripe pattern of window/mask, each 100 µm wide, exhibits an In*_x_*Ga_1−*x*_As composition distribution across the window, where the *x* value reaches its maximum at the window center and decreases towards the edges. This distribution is opposite to the commonly accepted distribution [17], which we believe is due to the conditions of our selective-area-epitaxy process, where the maximum concentration of In in the QW is achieved. As the concentration of the In precursor in the vapor phase increases, clusters enriched with In are formed on the surface. The formation of these InAs clusters results in reduced In content in the QW. In our case, the In precursor concentration in the vapor phase increases from the window center to the edge.

We also demonstrated that the curvature of the growth surface profile, resulting from variations in the thickness profile of the lower waveguide layer grown by selective area epitaxy, impacts the wavelength variation across the window during the growth of the QW under identical growth conditions. Up to a thickness of 0.6 µm in the central part of the window for the lower waveguide layer grown by selective area epitaxy, the wavelength changes smoothly from the center (982 nm) to the edge (992 nm). Upon further increase in the thickness of the lower waveguide layer, up to 1.9 µm, the wavelength changes very weakly across most of the window and only begins to increase sharply at a few micrometers from the edge, reaching 994 nm at the edge of the window.

Besides this, as can be seen from Figure 6, reducing *D*/*k* for solid solutions leads to the formation of a profile with greater curvature. On the other hand, as the window widens, the GRE value at the window center would decrease and approach 1, which corresponds to growth conditions during SE. This demonstrates additional possibilities for controlling the QW composition distribution across the window.

## Figures and Tables

**Table 1:** Description of experimental samples.

Parameter	SENQW	SEWQW	SAEWG1	SAEWG2	SAEWG3	SAEWG4
SE GaAs buffer layer	0.5 µm	0.5 µm	0.5 µm	0.5 µm	0.5 µm	0.5 µm
SE Al_0.3_Ga_0.7_As lower cladding	0.4 µm	0.4 µm	0.4 µm	0.4 µm	0.4 µm	0.4 µm
SE GaAs lower waveguide	0.4 µm	0.4 µm	0.3 µm	0.3 µm	0.3 µm	0.3 µm
SAE GaAs lower waveguide *	-	-	0.12 µm/ 3.57 min	0.6 µm/ 17.78 min	1.2 µm/ 35.58 min	1.9 µm/ 56.33 min
SE InGaAs QW	7 s	14 s	-	-	-	-
SAE InGaAs QW *	-	-	7 s	7 s	7 s	7 s
SE GaAs upper waveguide	0.4 µm	0.4 µm	-	-	-	-
SAE GaAs upper waveguide *	-	-	0.3 µm/ 8.88 min	0.3 µm/ 8.88 min	0.3 µm/ 8.88 min	0.3 µm/ 8.88 min
SE Al_0.3_Ga_0.7_As upper cladding	0.25 µm	0.25 µm	-	-	-	-
SAE Al_0.11_Ga_0.89_As upper cladding *	-	-	0.3 µm/ 7.85 min	0.3 µm/ 7.85 min	0.3 µm/ 7.85 min	0.3 µm/ 7.85 min

* For SAE layers, the thicknesses are from the window center and the growth time is indicated.

## Data Availability

Not applicable.
